# Diffusion/Reaction Limited Aggregation Approach for Microstructure Evolution and Condensation Kinetics during Synthesis of Silica-Based Alcogels

**DOI:** 10.3390/ijms24031999

**Published:** 2023-01-19

**Authors:** Nina Helena Borzęcka, Bartosz Nowak, Rafał Pakuła, Robert Przewodzki, Jakub Maksymilian Gac

**Affiliations:** Faculty of Chemical and Process Engineering, Warsaw University of Technology, 00-645 Warszawa, Poland

**Keywords:** cellular automata, condensation kinetics, methyltrimethoxysilane, diffusion/reaction limited aggregation, sol–gel transition

## Abstract

A base-catalysed methyltrimethoxysilane (MTMS) colloidal gel formation was implemented as a cellular automaton (CA) system, specifically diffusion and/or reaction-limited aggregation. The initial characteristic model parameters were determined based on experimental synthesis of MTMS-based, ambient-pressure-dried aerogels. The applicability of the numerical approach to the prediction of gels’ condensation kinetics and their structure was evaluated. The developed model reflects the kinetics properly within the investigated chemical composition range (in strongly reaction-limited aggregation conditions) and, to a slightly lesser extent, the structural properties of aggregates. Ultimately, a relatively simple numerical model reflecting silica-based gel formation was obtained and verified experimentally. The CA simulations have proved valid for understanding the relation between the initial chemical composition and kinetics constants of MTMS-based synthesis and their impact on secondary particle aggregation process kinetics.

## 1. Introduction

The sol–gel synthesis method is based on a transition between two colloidal systems. The transition may proceed due to hydrolysis, poly-condensation and re-esterification reactions [1,2,3]. The reactions occur, to a certain pH-dependent extent, concurrently [4,5]. It is a versatile method of producing many industrially and commercially important materials such as ceramics [6], glasses [7], coating films [8,9], adsorbents [10], insulation materials [11] and catalyst supports [3,12,13]. The chemistry of the transition strongly depends on the chosen precursors and the initial chemical composition of a reaction mixture. The final product’s structure—film, xerogel, aerogel, ceramic fibres, dense ceramics—depends on the chosen preparation and drying method [13]. 

During gel formation from organoalkoxysilane precursor molecules, three fundamental steps can be distinguished—the first two have already been mentioned: (i) hydrolysis and (ii) condensation, while (iii) is phase separation [2]. Phase separation was described by Issa and Luyt as a process when a reaction mixture loses its homogeneity to form a liquid in a continuous solid phase or a solid in a continuous liquid phase [2]. The first case is the definition of a gel, while the second is a sol or a precipitate (in the case of sedimentation). Mechanisms of phase separation for silica precursors were exhaustively studied by Nakanishi et al. [14,15,16]. The imposition of different chemical reactions and physical phenomena makes the investigation of organoalkoxysilanes formation kinetics a challenging yet necessary field of study. With such a variety of simultaneous factors that are impossible to investigate separately experimentally, the combination of modelling and experimental studies could provide an in-depth understanding of the fundamentals of the sol–gel transition. Studies on gel formation kinetics are also an essential basis for process optimisation.

Currently, there is plenty of ongoing research regarding the development of novel materials for very specific applications, such as glasses [17,18], aerogels [19,20,21,22], batteries [23,24], and electrodes [25,26,27,28,29,30], indicating a high commercialisation potential. A significant aspect that certainly affects a product’s properties is structure formation kinetics. 

There are five basic approaches for theoretical sol–gel representation: (i) a method based on the Smoluchowski equation [31,32,33], (ii) a Brownian dynamics approach [34,35], (iii) Langevin dynamics [36,37,38], (iv) percolation theory [39,40], and (v) diffusion or reaction limited particle or cluster aggregation (DLA/DLCA/RLA/RLCA) [41,42]. 

Every method is associated with different limitations. The Smoluchowski equation does not provide structural information and is limited to a kinetics description [43]. Brownian and Langevin dynamics require a knowledge/designation of peculiar physicochemical parameters (based on our experience with coarse-grained molecular dynamics studies of polyelectrolytes’ pH-dependent conformations [44]). Finally, percolation theory and DLA/RLA models were initially recognised as approaches that provided a good view of the internal structure but no direct information about system evolution kinetics [31,41]. However, this disadvantage of DLA/RLA was overcome, e.g., by Hsieh et al. [41] and in our previous work [41,45].

**Figure 1:**
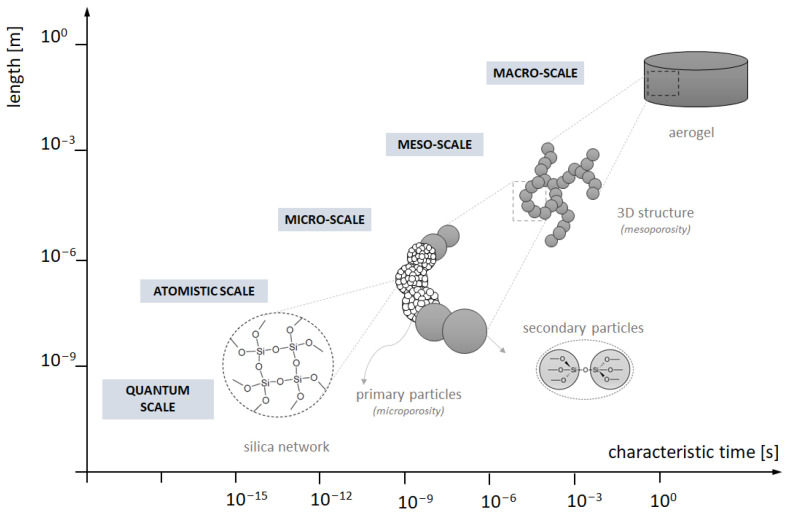
Compilation of hierarchic structure levels and scales in terms of numerical modelling.

These approaches can be implemented via different models of computation, depending on the chosen scale (Figure 1), such as the Monte Carlo method [46], molecular dynamics (reactive, coarse-grained) [1,36,37,38,47,48,49], population balance [50] and cellular automata. The last one is rarely applied for gelation description [42], though is more common for investigating mechanical behaviour [51,52]. However, cellular automata are used for many processes concerning aggregation due to diffusion (possibly with chemical reaction) [53], such as recrystallisation [54] or flocculation [55].

The most significant challenges associated with modelling studies are compliance with experimental observations, model utility and scope of applicability. In the presented paper, we focused on providing an approach to the translation of the synthesis parameters of methyltrimethoxysilane (MTMS) gels on characteristic parameters in a developed 3D DLCA/RLCA cellular automaton. CA is a well-known method, although most studies lack any thoughts on particle/cluster connection probability and only concern diffusion-limited aggregation [41,42]. The presented work focuses on the interpretation of this parameter and finding the relation between experimental determinable values, which is the main novelty advancing the state of the art.

## 2. Results

### 2.1. Estimation of Model Parameters 

**Figure 2:**
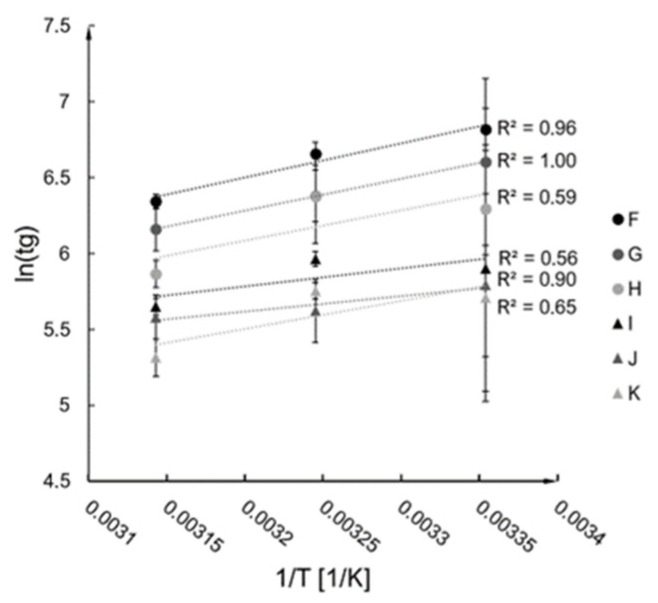
Dependence of gelation time on temperature.

**Figure 3:**
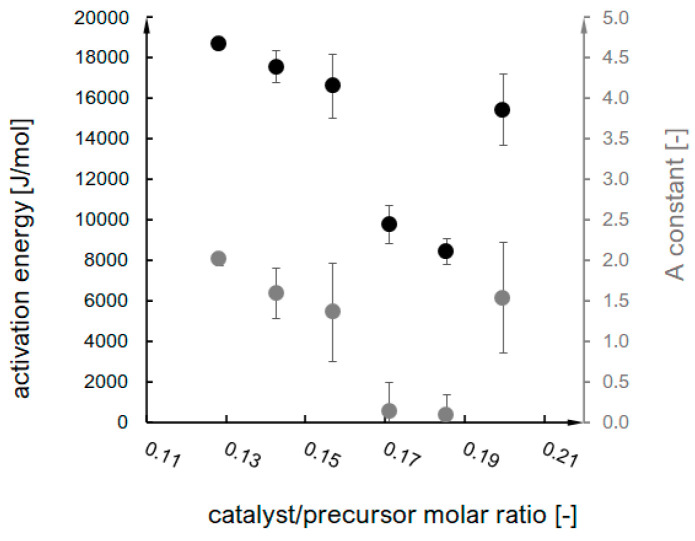
Designated values of $E_{a}$ and A constant as catalyst/precursor molar ratio function.

Activation energy E and A constant were established based on the conducted measurements of gelation time for A–F syntheses for the chosen temperatures (described in Section 4.1). The results were plotted in $\ln\left(t_{g}\right)=f\;\left(1/T\right)$ form and presented in Figure 2, along with the fitted linear trendlines (R^2^ values are presented in the graph). According to the linearisation of the Arrhenius equation (Equations (1) and (2)), the trendline constants allowed for an estimation of the E and A values for the investigated syntheses (presented in Figure 3).

(1) $$k=A_{A}\exp\left(-\frac{E}{RT_{g}}\right)$$

(2) $$\ln t_{g}=A+\frac{E}{RT_{g}}$$

A denotes the Arrhenius constant, R—gas constant, and E—activation energy. A is a constant dependent on the Arrhenius constant. The A constant can be interpreted as the number of molecular collisions with proper orientation, and the exponential element $\exp\left(-\frac{E}{RT_{g}}\right)$ as the probability of a successful collision; therefore, k is the number of effective molecular collisions per second.

**Figure 4:**
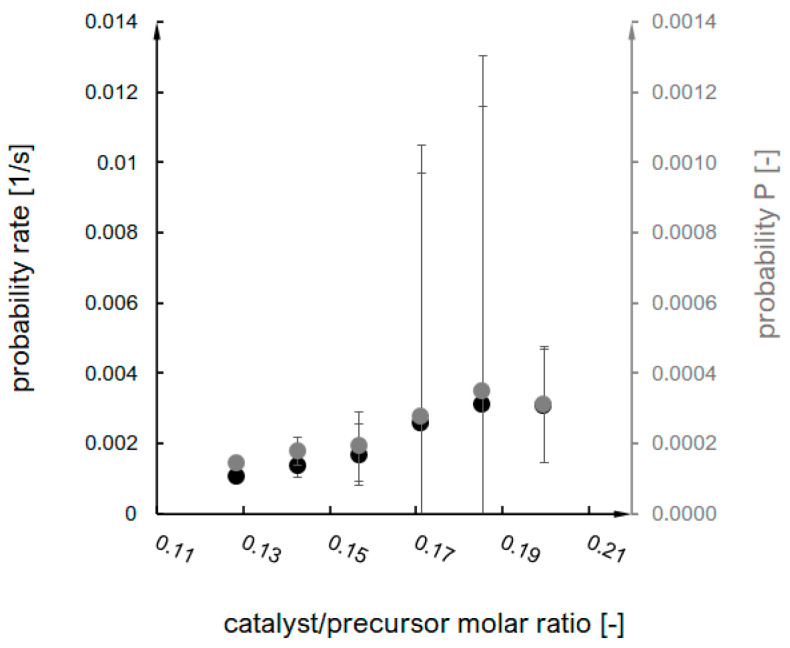
Dependence of probability and probability rate function on catalyst/precursor molar ratio.

These values were a basis for the probability parameter designation (according to the interpretation of the Arrhenius equation), which is an equivalent of an effective collision probability value and the primary model parameter within the presented research. The probability rate was designated directly based on the Arrhenius equation, while probability P was multiplied by the time step value (characteristic for each synthesis) (Equation (4)). The probability and probability rate dependence on the catalyst/precursor molar ratio are shown in Figure 4.

(3) $$P=\left[A\cdot \exp\left(-\frac{E}{RT_{g}}\right)\right]dt$$

Timestep for each case was calculated on the basis of our previous research; we have experimentally measured (using the tilting test-tube method) the value of gelation time, and we can interpolate a predicted numerical value of a gel-point based on the interpolation of 25 studied cases.

The cellular automaton requires two set parameters: (i) secondary particle concentration and (ii) probability of an effective collision.

Within the numerical studies, a set of 25 cases was simulated: $c=\left\{0.5,\;1,\;2,\;3,\;4\;\left[{\%}_{vol}\right]\right\}$ and $P=\left\{10^{-4},\;10^{-3},10^{-2},10^{-1},1\;\left[-\right]\right\}$. For example, a concentration of 1% means that secondary particles occupy 1% of cells. As to the probability value, P=1 means there is a 100% probability that two adjacent particles (located in adjacent cells, according to Moore’s neighbourhood) will become an aggregate (thus, the DLA mechanism is responsible for the aggregation). For P≪1, most collisions do not end in a permanent association of particles/aggregates (RLA mechanism).

Subsequently, the experimental conditions had to be “translated” into model parameters. The first step was an estimation of the secondary particle concentration in a sample. Knowing the volume of an alcogel, the mass of the skeleton (aerogel mass), and structural density, the volume concentration of secondary particles in a system was calculated according to Equation (1):

(4) $$c=\frac{V_{sp}}{V_{a}}=\frac{n_{sp}V_{sp1}}{V_{a}}=\frac{m_{s}}{m_{sp}}\frac{V_{sp1}}{V_{a}}=\frac{m_{s}}{V_{sp1}\cdot \rho_{s}}\frac{V_{sp1}}{V_{a}}=\frac{m_{s}}{\rho_{s}V_{a}}\left[{\%}_{vol}\right]$$

where $V_{sp}$ is the volume of secondary particles, $V_{a}$ is the volume of alcogel, $n_{sp}$ is thenumber of secondary particles in the system, $V_{sp1}$ is the volume of one particle, *m_s_* is the mass of skeleton (aerogel mass), and $\rho_{s}$ is structural density ($1.38\;\left[g/{\mathrm{cm}}^{3}\right]$).

Probability was evaluated based on the Arrhenius equation, but recounted per timestep *dt*, because this value is the most adequate to the model parameter (Equation (3)).

Timestep for each case was calculated on the basis of our previous research; we have experimentally measured (using the tilting test-tube method) the value of gelation time, and we can interpolate a predicted numerical value of a gel-point based on the interpolation of 25 studied cases.

All of the designated model parameters, *c*, *P* and *dt* for actual experimental syntheses A–F, are gathered in Table 1.

### 2.2. Kinetics Analysis 

While analysing the kinetics curves, three phases can be distinguished. The first one refers to nucleation, which happens on the molecular level, and does not cause the turbidity increase. The solution is clear, and mass growth is negligible. The second phase is visible as an intensive increase in absorbance value that can be correlated with silica skeleton formation via siloxane bonding. A plateau informs us of reaching the gel point where gel is in dynamic equilibrium—gel can be reorganised via coarsening and Ostwald ripening. 

Within the model, the first phase (nucleation) cannot be observed in our numerical system—particles in CA are already formed, and their aggregation leads directly to the second phase. The third phase, regarded as a plateau, is when mostly aggregate clusters that were already detectable impact the structure formation process and morphology, but does not translate into an increase in the mass of the condensing product.

**Figure 5:**
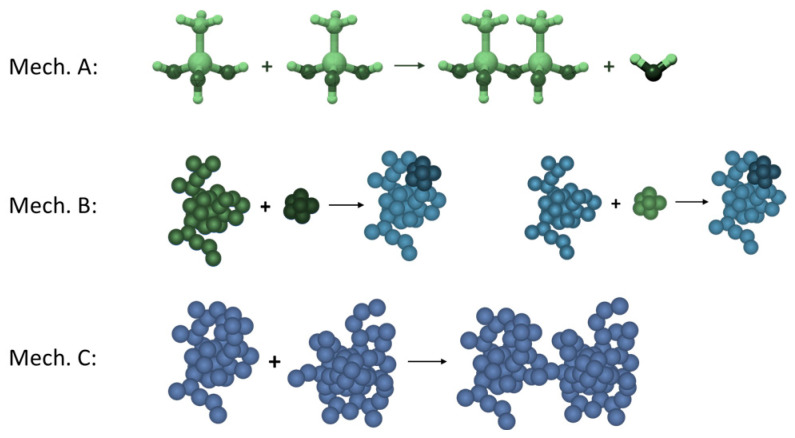
Basic condensation mechanisms. Green structures are undetectable by measurements. Blue structures are big enough to be registered.

A summary of these theoretical considerations and assumptions is gathered in Table 2. We identified condensation mechanisms in each of the distinguished phases, according to Figure 5. During the first phase, the mass of the condensing product does not (or barely) increase. It means that the hydrolysed precursor molecules’ condensation leads to nuclei formation, but they are too small to be detected by UV-Vis spectrophotometer. During the second phase, we have intensive mass growth. It means that large aggregates are becoming rapidly detectable. A mechanism that would explain such phenomena is bonding two undetectable aggregates into a bigger one which is immediately registered. Another option (however, less rapid) is when a small undetectable aggregate bonds with a detectable one. It is less rapid because the total detected mass would increase by one unit, but when two bigger (but not yet detectable) aggregates suddenly merge into a bigger one, the detected mass thus increases in step response. During the third phase, the mass is barely increasing because most of the clusters are big enough to be seen, and when they bond with each other, it does not impact mass growth, only structure formation and morphology.

**Figure 6:**
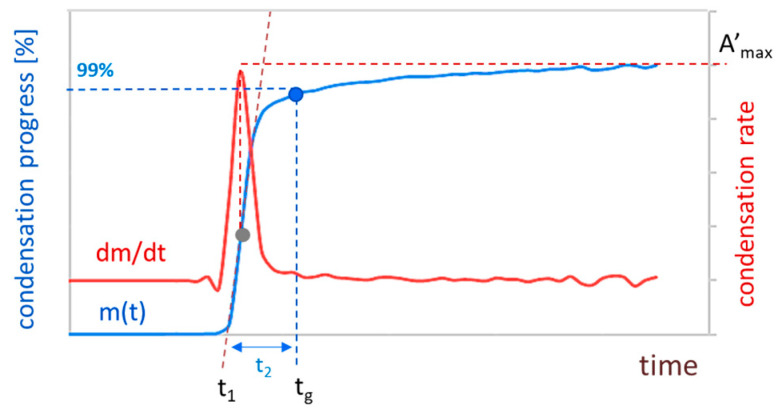
Scheme for quantitative analysis of condensation kinetics.

The above qualitative considerations were quantified to compare and precisely analyse kinetics. The obtained results were interpolated by the spline method and differentiated. As described in our previous research [45], condensing skeleton mass is directly proportional to the absorbance value. Thus, we use a normalised absorbance value ($A\left(t\right)/A_{max}$) and identify its changes as condensation progress in relation to the final aerogel mass ($A_{max}$). The red line in Figure 6 is the first derivative (dm/dt), and it gives information about condensation rate evolution during the whole process. It also provides a specific and easy-to-determine point—the time when the maximum reaction rate is achieved, marked on dm/dt (blue line) as a grey dot. By making a tangent line to this point, it is possible designate the assumed time of the nucleation phase $t_{1}$. Another point that we can quantify is the gel point. In our previous works, we observed that for particle-aggregate structures (nucleation and growth mechanism), the gel point $t_{g}$ was approximately when the normalised absorbance was equal to 99%. We assumed this point as the beginning of the third phase—the plateau. The period between these two points is the time of the second phase $t_{2}$. 

**Figure 7:**
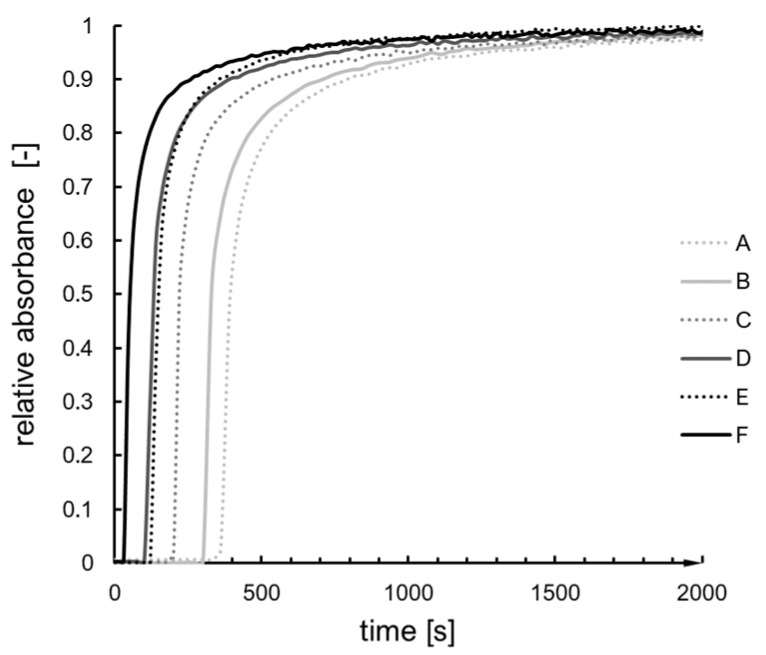
Condensation kinetics curves of A–F syntheses.

**Figure 8:**
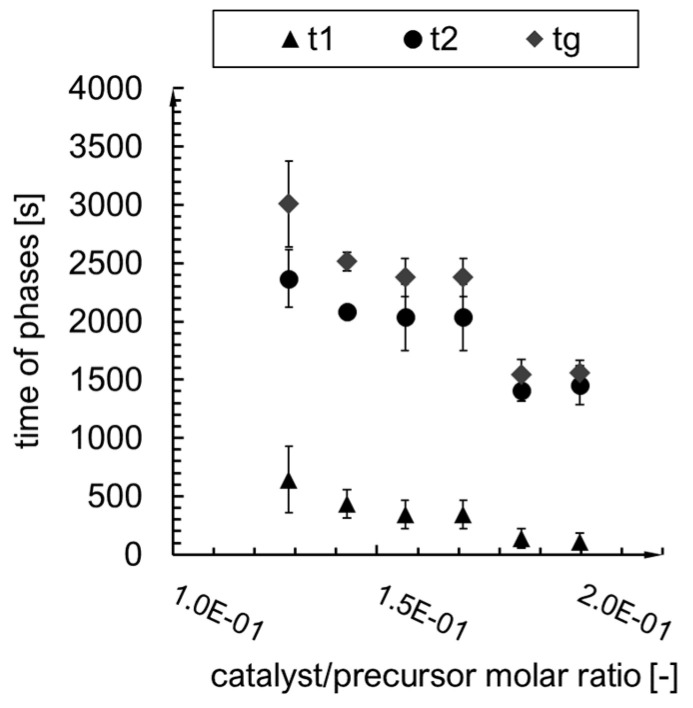
First phase, second phase and gelation time for the A–F syntheses.

**Figure 9:**
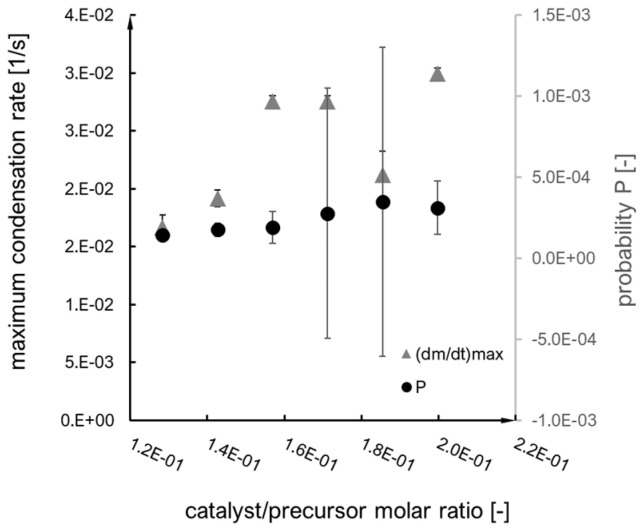
Dependence of probability and maximum condensation rate (*dm*/*dt*)*_max_* on base/precursor molar ratio.

**Figure 10:**
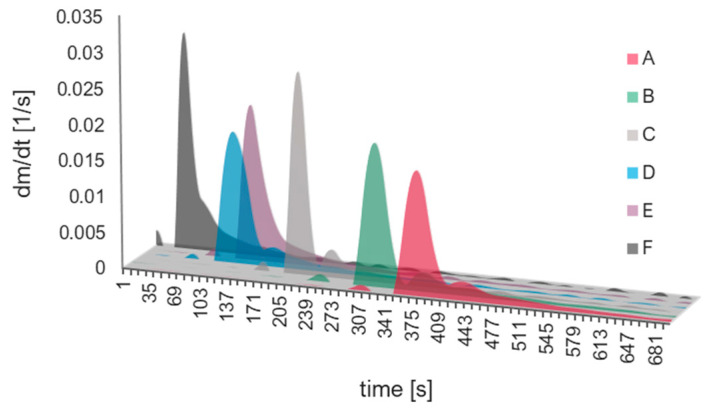
Dependence of *dm/dt* on time of reaction for syntheses A–F.

The curves obtained experimentally (Figure 7) are sigmoidally shaped, with a short period of the first phase compared to the second one (Figure 8). With the increase in the catalyst/precursor molar ratio, the gelation time decreases rapidly (A–D syntheses), then more slowly (E, F syntheses). When we look at the maximum value of the first derivative (condensation rate) (Figure 9), one can notice a drop for D and E syntheses. The dependence of dm/dt on time or reaction is presented in Figure 10. We can assume that three factors connected with dm/dt have an impact on the gelation time value:

i. The value of the maximum condensation rate ${\left(\frac{dm}{dt}\right)}_{max}$;
ii. The time corresponding to the occurrence of the maximum rate;
iii. The “span” of the first derivative’s peak.

Thus, even though the value of the first derivative is not constantly increasing, we observe that gelation time decreases with a higher contribution of catalyst in the system, presumably due to the imposition of the three (i–iii) factors. However, due to data interpolation accuracy, the first derivative seems to be the most reliable value that can be obtained numerically.

**Figure 11:**
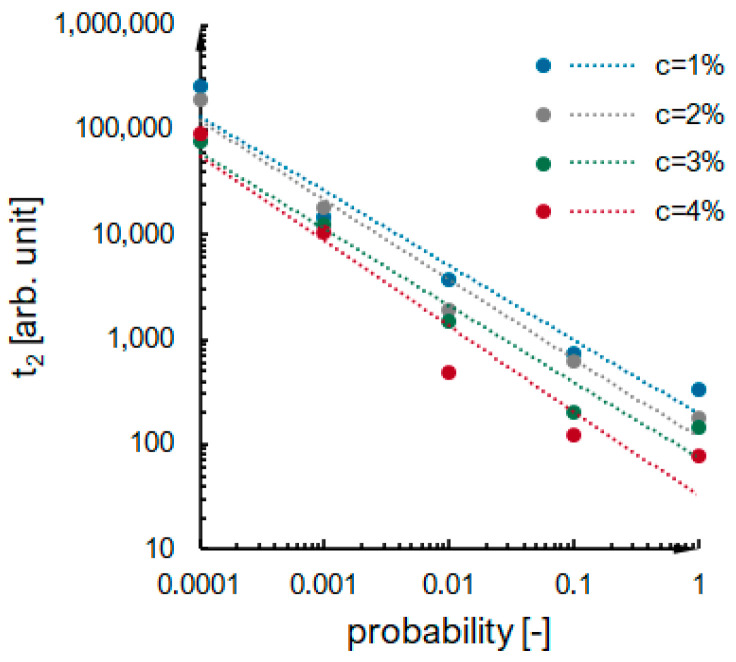
The dependence of the second phase duration on effective collision probability for variable concentrations of secondary particles in a system.

**Figure 12:**
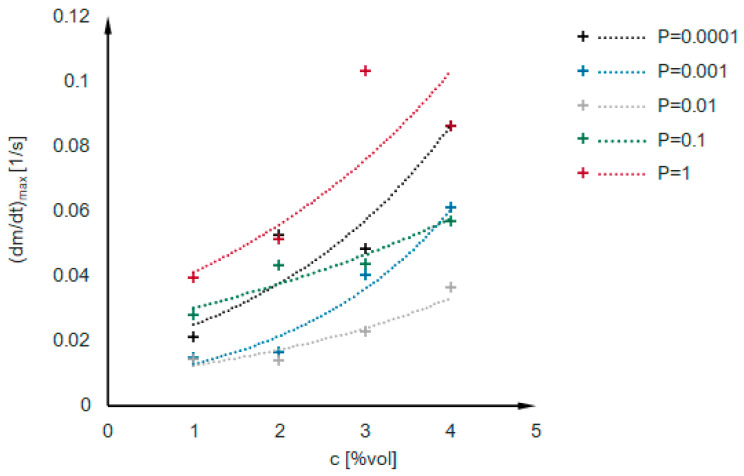
The values of maximum condensation rate obtained for numerical simulations.

**Figure 13:**
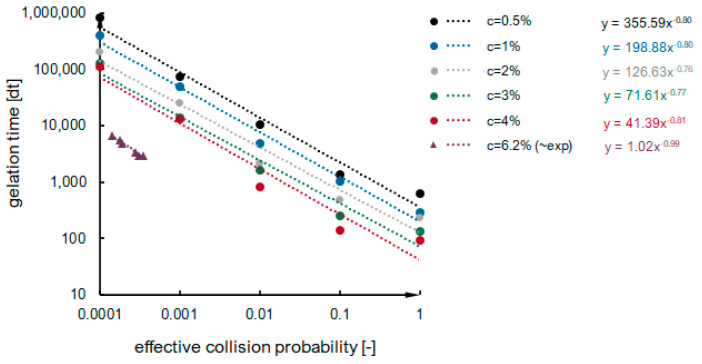
Gelation time dependence on effective collision probability for different secondary particle concentrations (dots—numerical simulation results, triangles—experimental results).

Kinetics curves obtained numerically for the mentioned 25 cases are presented in Appendix B. There is no first gelation phase, as nucleation is not considered during simulations. The dependence of the second phase duration on effective collision probability for variable concentrations of secondary particles in a system is presented in Figure 11. The dependencies have a power function character, shown as dotted lines. The values of the maximum condensation rate obtained for numerical simulations are presented in Figure 12. One can notice that the maximum condensation rate increases with increased particle concentration. It can be explained by referring to the mechanisms presented in Figure 6—the more particles we have, the more clusters will be created simultaneously, and a higher increase in reaction rate will be observed once the clusters reach the detectable threshold mass. However, there is no constant manner in the presented relation—the numerical kinetics curves are not as smooth and applicable for interpolation and differentiation as experimental curves obtained with low measuring time interval values. However, when looking globally at the numerical results (Appendix B, Figure 11 and Figure 13), we see that reaction proceeds faster when:

i. The probability of an effective collision increases;
ii. The concentration of secondary particles increases.

In Figure 13, numerical and experimental results of gelation time were plotted. Numerical $t_{g}$ values were designated as described in Section 2.3 as the point when the structure is 99% interconnected. Experimental $t_{g}$ values (triangles on the graph) were measured using the tilting test tube method but recalculated as a number of the time step so that it would be comparable with the numerical results (dots). To achieve that, the established *dt* values (Section 2.1) were used $t_{g}\;\left[dt\right]=t_{g}^{tilting}/dt$.

### 2.3. Verification of Kinetics

Having in mind all of the adopted simplifications and assumptions within the model (discussed in the following Section 3), we attempted to compare the shape of the kinetics phase with a focus on the second phase of gelation, because it is the only phase that occurs for both the numerical and experimental system, and we can define the length of the phase based on the kinetic curve shape in a precise and repeatable way. We assumed that the duration of this phase is related to the maximum value of the condensation rate ${\left(\frac{dm}{dt}\right)}_{max}$. The ratio ${\left(\frac{dm}{dt}\right)}_{max}\;/t_{2}$ was calculated on the basis of experimental results and estimated for numerical data by extrapolating results for higher concentrations $6.2\;{\%}_{vol}$ and interpolated for the probability of an effective collision per time step included in Table 3.

**Figure 14:**
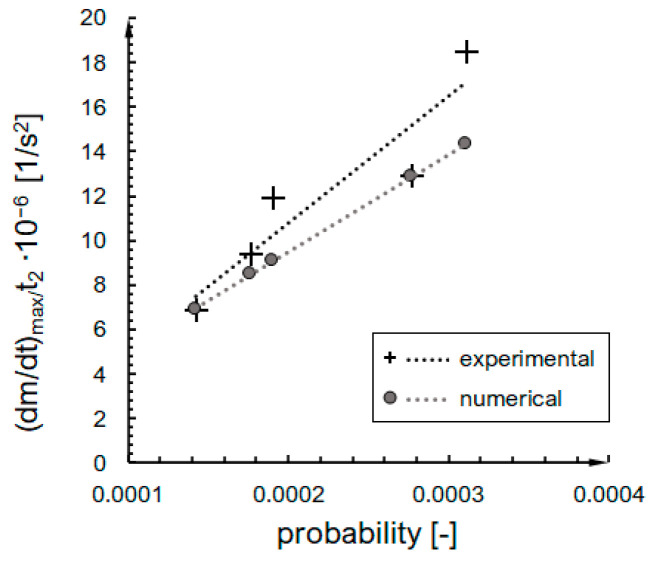
The dependence of (*dm*/*dt*)*_max_*/*t*_2_ ratio on probability of an effective collision for experimental and numerical approach.

The results are presented in Figure 14. For the examined RLCA range, the ratio ${\left(\frac{dm}{dt}\right)}_{max}/t_{2}$ depends linearly on the probability of an effective collision with convincing coefficients of determination ($R^{2}=0.87$ for experimental and $R^{2}=1$ for numerical trendline). The formulas of the trendlines are presented in Table 3. The slope factors are very similar—the a parameter for a numerical line is only 23% less than the experimental one. The difference for constant terms is higher—the value of numerical b is positive, and the experimental is negative. Nevertheless, the disparities do not seem as significant when the linear character of the dependencies and the order of magnitude are the same.

### 2.4. Structure

**Figure 15:**
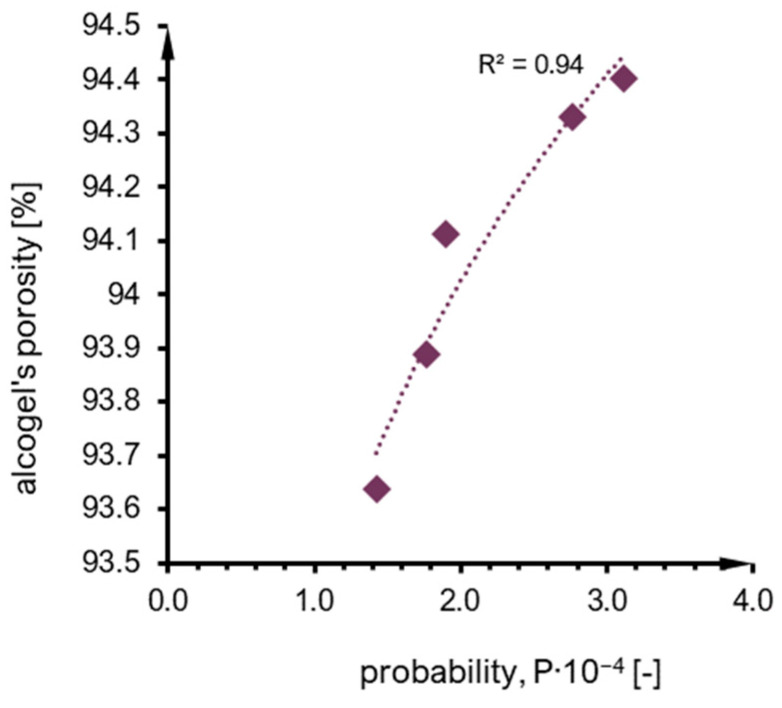
An alcogel’s porosity due to probability value—results for experimental syntheses.

**Figure 16:**
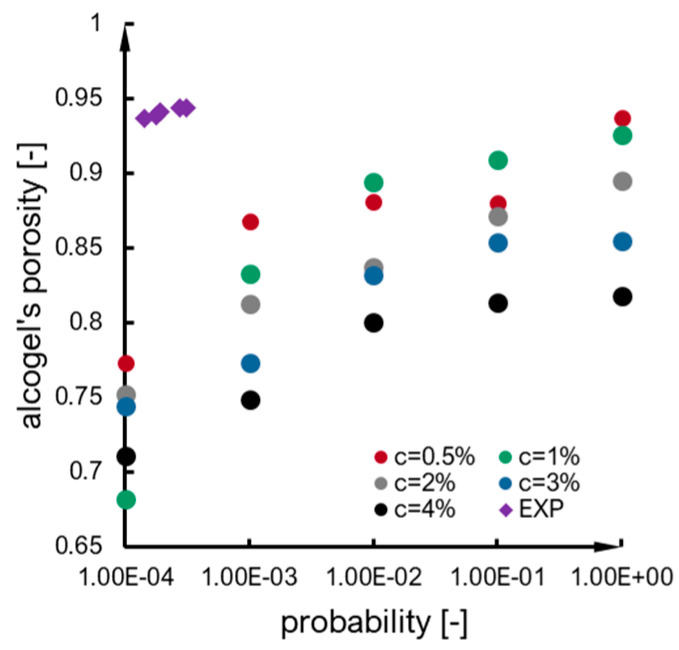
An alcogel’s porosity due to probability value—numerical vs. experimental results.

**Figure 17:**
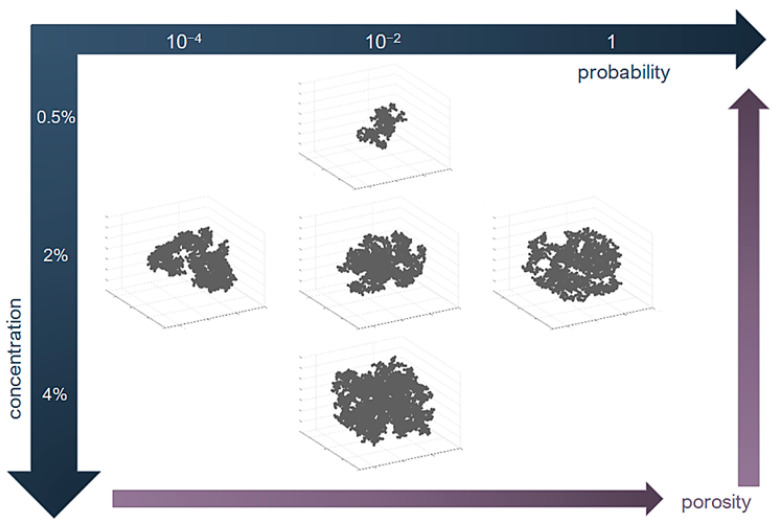
Overall scheme presenting relations between particle concentration, probability of effective collisions and structure properties: morphology, fractal dimension and porosity.

The porosity of alcogel samples (A–F syntheses) is presented in Figure 15 as a function of probability calculated based on the interpretation of the Arrhenius equation. The tendency can be approximated as a power function with a moderately fair $R^{2}$ value. The main observation is that porosity mainly increases along with probability value. One of the samples (E) could not be taken into account because of the exceptionally high measurement errors of calculation of probability (as presented in Figure 10). A similar observation regarding the porosity–probability relation was for numerical results (Figure 16), which can also be approximated by a power function. However, when the results are compared together (numerical vs. experimental), it can be noticed that alcogel porosity measured experimentally is much higher than that predicted via simulation results. The overall scheme presenting relations between particle concentration, probability of effective collisions and structure properties (morphology and porosity) is included in Figure 17. One can see how the structure morphology changes with model parameters (concentration and probability). Naturally, with a concentration increase, an aggregate becomes bigger. With increased probability, the structure tends to become more branched (DLCA structures) compared to more dense structures obtained for low probabilities (RLCA). 

All of the obtained structures for the studied numerical cases are presented in Appendix A.

## 3. Discussion

The goal of the paper was to compare the experimental and numerical curves quantitatively. However, an awareness of several disparities in the experimental and numerical approaches should be raised. 

Within the numerical system, the initial size distribution of secondary particles is extremely monodisperse. The second phase of condensation proceeds, as it was subsequential to nucleation. Furthermore, the reaction is irreversible; no bond/interaction breaks within the already-created aggregate. 

In the experimental system, the stages are not strictly subsequential in the same simple manner as in the cellular automaton. The borders can be indistinct for reactions that can occur parallelly or reversibly (hydrolysis, condensation) and for condensation phases (nucleation/primary particle formation, secondary particle aggregation). Massive aggregates can sediment. The experimental system is very complex, and the process cannot be observed in vivo from the very start (molecular level). 

However, despite the assumed simplification at a certain level, results can be considered satisfactory and complementary to the current state of the art. The estimated E values look consistent with the literature data for different organoalkoxysilane precursors (comparison is presented in Table 4).

The research indicates that DLCA/RLCA model can be used for aggregation kinetics investigations. Furthermore, it was proved that experimentally measurable values for a certain synthesis, such as the time of gelation and alcogel porosity (or secondary particle diameter), can be translated into model parameters, such as the probability of an effective collision, particle concentration within a numerical system and time step of a simulation. According to our knowledge, such a “translation” of experimental values into numerical parameters was not yet described in the literature. 

The similarity of the kinetics character shown in Figure 14 presents a very narrow range of probability values. According to our calculations, it is a very strong RLCA regime with a probability range $\langle 1.4\times 10^{-4},\;3.1\times 10^{-4}\rangle$. The P range investigated within our cellular automaton studies $=\langle 1\times 10^{-4},\;1\rangle$. Thus, discrepancies between tendencies observed for experimental and numerical systems can be observed. They were gathered and described in Table 5.

The porosity comparison of structures obtained experimentally and numerically is not very satisfactory. The main tendency is the same (an increase in porosity along with the value of an effective collision probability); however, the values are underestimated—alcogel porosity measured experimentally is much higher than could be predicted based on simulation results. These aspects certainly should be improved in the future.

## 4. Materials and Methods

### 4.1. Experimental

The synthesis method was the two-step, acid–base sol–gel synthesis as described by Rao et al. [57]. The reaction mixture was prepared by mixing the proper volume of metyltrimethoxysilane (MTMS, purchased from Sigma-Aldrich, St. Louis, MO, USA) as a silica precursor, methanol (MeOH, Stanlab, Lublin, Poland) as a solvent and an aqueous solution of oxalic acid (concentration 0.01 M, Sigma-Aldrich) as an acidic catalyst to initiate the hydrolysis reaction. After a suitable time for this reaction to occur, an aqueous solution of ammonia (1 M, Eurochem BGD, Tarnów, Poland) was added to the mixture as the base catalyst of the gelation reaction. As a result, the alcogel was obtained. Six samples were chosen for the condensation kinetics investigation. All samples were prepared to have a constant MTMS/MeOH ratio (vol. 1:2) and a variable volume of aqueous catalyst solutions. Chemical compositions are presented in Table 6.

**Figure 18:**
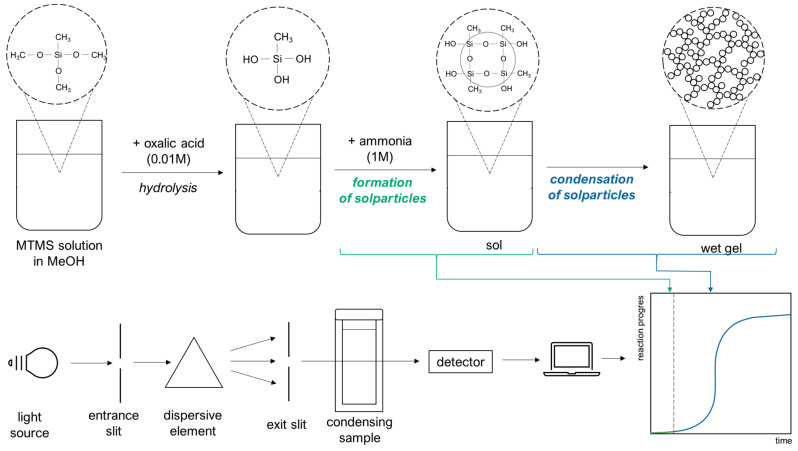
Scheme of MTMS alcogel synthesis with parallel spectrophotometric measurements of condensation kinetics.

The kinetics of gelation were investigated using measurements of UV-Vis light absorbance during the condensation process (scheme presented in Figure 18), utilising a Genesys 10 S UV–Vis Spectrophotometer Thermo Scientific with a wavelength λ=633 [nm]. Changes in the absorbance of the mixture were found to be proportional to the mass of the formed alcogel, which was justified in our previous work [45].

Besides the changes in absorbance, the gelation time was measured to evaluate kinetic parameters in the Arrhenius equation. We used the basic, although easy, and commonly used tilting test-tube method [40,58,59,60]. The measurement for each sample was performed three times and averaged for the three chosen temperatures of gelation (25, 35 and 45 °C). We used the analogical assumption, as in our previous work [61], that the gelation time is inversely proportional to the mean (or initial) reaction rate and, thus, to the reaction rate constant (Equations (1) and (2)).

### 4.2. Numerical Model Description

The three-dimensional computing domain, divided into a regular grid of cells, represents investigated system. We investigated methyltrimethoxysilane (MTMS) gels with a “particle aggregates” type of structure, according to Nakanishi’s division [14]. It is a hierarchical structure where precursor molecules undergo poly-condensation reactions and form primary particles that subsequently aggregate into secondary particles [62]. Thus, the model represents DLCA/RLCA mechanisms (cluster aggregation).

In our system, we assume that phase separation has already occurred due to nucleation and growth mechanism, and there is a certain amount of secondary particles. The initial conditions of a simulation (location of secondary particles) are pseudo-randomly generated according to a set concentration value $c=\left(N_{occupied}/N_{cells}\right)\cdot 100\%$ (where $N_{occupied}$ is the number of secondary particles and $N_{cells}$ is number of cells within the computing domain). According to the cellular automaton definition, each cell is described by one of a finite number of states. There are only two possible states for each cell of the automaton—a cell can be occupied by a solid or a liquid phase. 

Generally, in cellular automata systems with aggregation, we can distinguish three probability values determining the system dynamics: (i) the movement/diffusion probability $P_{D}$, (ii) the reaction (effective collision) probability P, and (iii) the breaking probability $P_{B}$ [63]. 

The diffusion probability was determined based on the Stokes–Einstein equation for the diffusion of spherical particles (D~1/r), which indicates that the diffusion coefficient is inversely proportional to the size of a particle. Thus, $P_{D}\sim 1/r$, where r is the radius of an aggregate (assumed to be a sphere), and $N_{a}$ is the number of secondary particles within the aggregate.

(5) $$P_{D}\sim \frac{1}{r}$$

**Figure 19:**
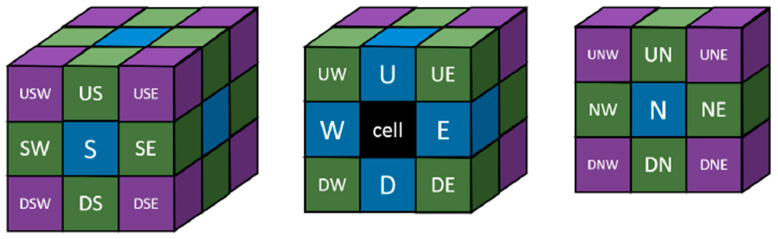
Graphical representation of Moore’s neighbourhood for the 3D CA; each of the letters corresponds to directions: North, South, East, West, Up, Down with reference to the black cell.

A collision occurs when two particles meet in adjacent cells (according to Moore’s type of neighbourhood presented in Figure 19). However, in general, not every collision leads to the permanent attachment of the particle to an aggregate or two aggregates to each other. For this to take place, apart from the collision, a surface reaction followed by the formation of a “neck” must also occur. Such a collision is called as an effective one. The effective collision probability was determined based on the interpretation of the Arrhenius equation. To evaluate the Arrhenius constant and activation energy, it was assumed that the gelation time $t_{g}$ was inversely proportional to the reaction rate k, thus $t_{g}\sim 1/k$.

Thus, the time of gelation can be expressed in the form of Equation (2), where A is a constant dependent on the Arrhenius constant. This representation of the Arrhenius equation allows the establishment of A and E values [13,56], thus the reaction probability for a specific experimental synthesis. More information on the evaluation of model parameters is presented in the following sections. If the reaction between adjacent particles does indeed occur, these particles start to act as one aggregate (particles that belong to different aggregates may also react with one another).

The last parameter $P_{B}$ is the probability of breakage between adjacent, connected particles, although this aspect is not taken into a consideration within the presented model.

### 4.3. Kinetics Simulation

As mentioned in Section 1, DLCA/RLCA modelling was not originally intended to investigate the system’s kinetics but to study the structure formation. In our case, it was the main objective to develop an algorithm that would be analogical and comparable with experimental measurements. Therefore, we focused on the mechanism of spectrophotometric measurement. During gelation, at first, the reaction mixture is transparent. As the reaction progresses, clusters of repeating units are formed, and when they reach a certain, not yet quantified size, they appear to be visible in the UV-Vis spectrum. The sample gradually becomes turbid, causing an increase in the sample’s absorbance value. 

**Figure 20:**
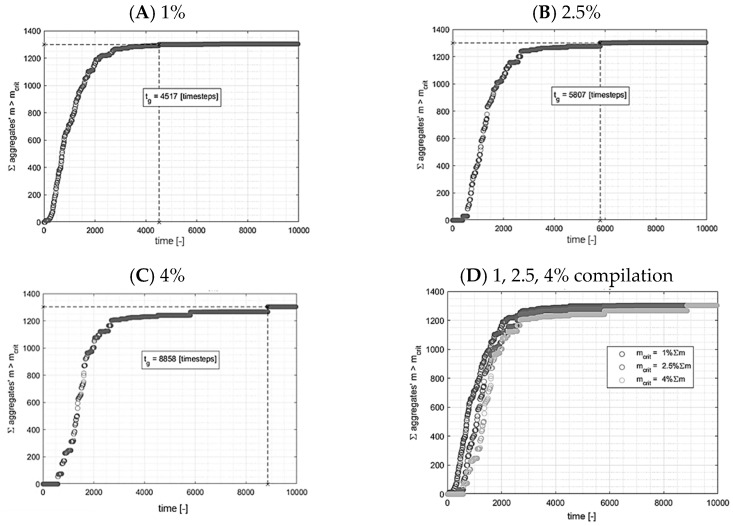
Impact of an aggregate threshold size on condensation kinetics curve.

In the presented numerical system, when an aggregate reached a detectable size (i.e., consisted of a set number of particles), its mass was added to the total sum of the condensed product and, thus, the condensation kinetics curve increased. An important aspect was defining a detectable size to correlate properly with experimental measurements. Three values were chosen to be tested: (i) a detectable size equal to 1% of a total number of secondary particles, (ii) equal to 2.5%, and (iii) equal to 4%. This approach for detectable size definition allows for the scaling of the parameter due to the concentration value or computing domain size. The kinetics curves registered for these three cases are presented in Figure 20A–C and an overall comparison is shown in Figure 20D. Referring to our previous studies [45], a gelation point for previously investigated synthesis was observed when absorbance reaches approximately 99% of its maximum value, and the gel point is usually located right after the inflexion of a curve (presented in Figure 6a and included in reference [45]). Considering all these facts, the detectable size was chosen as 1% of a system’s total number of secondary particles. One should be aware of the arbitrary character of this parameter. However, it seems as a good initial assumption. The kinetics designation procedure continued until all particles were connected to form one big aggregate.

### 4.4. Structural Analysis

**Figure 21:**
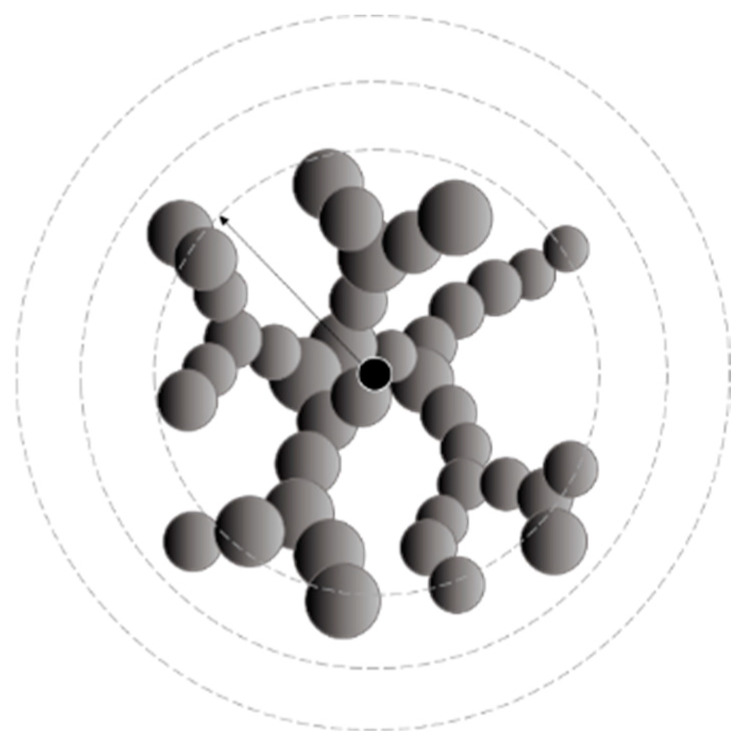
Scheme of porosity designation.

**Figure 22:**
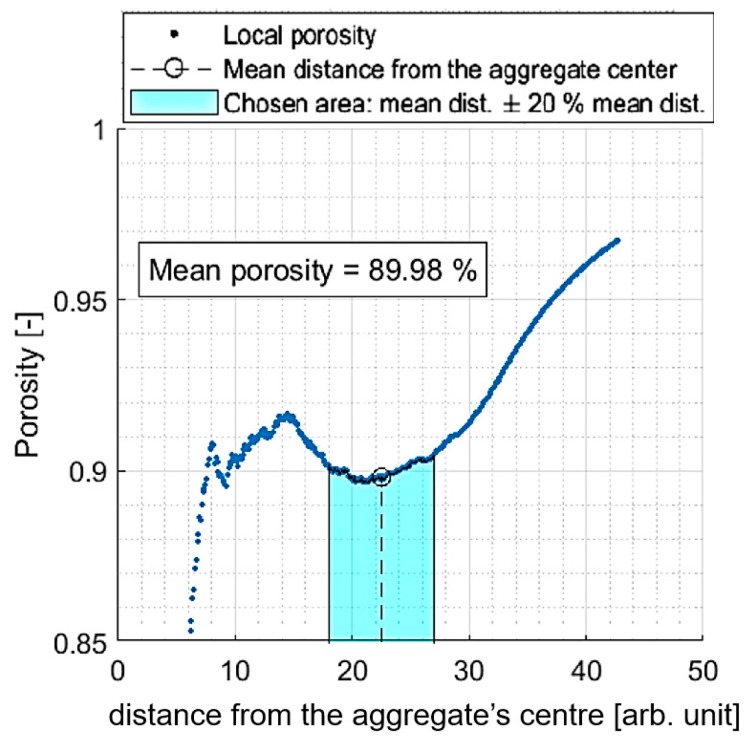
Scheme of porosity designation. Exemplary plot presenting local porosity dependence on distance from the aggregate’s centre and the chosen representative area.

The obtained structures were studied in terms of their porosity ε, determined as the number of empty cells within a volume of a certain sphere (which is centred in the aggregate’s centre of gravity) (Figure 21). However, some inaccuracies had to be taken into account, including the fact that porosity is underestimated in the regions close to the centre of the aggregate (in particular, if we chose a distance smaller than the radius of a single particle, we would obtain a result of 0). At the same time, this value may be overestimated for greater distances from the centre, where the aggregate goes smoothly into empty space (there is no strict boundary, let alone a sphere-shaped boundary). Therefore, we needed to select a representative area in the aggregate under consideration. We chose the mean distance of each particle to the aggregate’s centre as a reference point as it could be easily and precisely determined and because the local porosity value seemed approximately constant around this point. We assumed our representative area for porosity designation would be $\langle 80\%\;\bar{r},\;120\%\;\bar{r}\rangle$. An exemplary plot presenting local porosity dependence on the distance from the aggregate’s centre and the chosen representative area is presented in Figure 22.

## 5. Conclusions

This paper successfully attempts to translate experimental synthesis conditions into CA model parameters in terms of kinetics curve comparisons. 

Time of gelation measurements were used for A and E designation and subsequently for estimating the effective collision probability. 

Alcogel porosity (or measurement of mean secondary particle diameter) is applicable for estimating particle concentration in the numerical system.

Gelation time can also be used to approximate the real value of a time step.

According to our knowledge, such a “translation” of experimental values into numerical parameters had not yet been described in the literature, and we believe it can advance the current state of the art.

The comparison of kinetics curve shape is satisfactory. The ratio ${\left(\frac{dm}{dt}\right)}_{max}/t_{2}$ depends linearly on the probability of an effective collision for both experimental and numerical results. The slope factor difference is 10.1%, and the constant term difference is 22.8%. Nevertheless, the disparities seem acceptable, as the linear character of the dependences order of magnitude is the same.

Regarding the structure of aggregates, the main relation between alcogel porosity and probability is the same for both experimental and numerical results: an increase in porosity along with the effective collision probability value. However, we do not consider the quantitative comparison as satisfactory and it should be improved in the future.

The comparison of structures obtained experimentally and numerically is not as satisfactory. The main tendency is the same (an increase in porosity along with the effective collision probability value). However, the values are underestimated—alcogel porosity measured experimentally is much higher than could be predicted based on simulation results. These aspects certainly should be improved in the future.

Two possible improvements would minimise the simplification level of the model and should be discussed. The first one is the determination of gel point—it could be defined as a point when aggregates cannot rotate without hindrance. It would be analogical to the tilting test tube measurement method in some way, and it should be more precise than our simplified method. The second improvement would be considering the parallelism of primary and secondary particle formations. It could be completed using DLCA/RLCA aggregation and simultaneously adding new particles to the system.

To summarise, base-catalysed colloidal gel formation was successfully implemented as a CA model. The applicability of the numerical approach on the prediction of gels’ condensation kinetics and their structure was evaluated, and it seems reliable within a strong RLCA regime. The developed model reflects the kinetics properly within the investigated chemical composition range. The structure analysis needs improvement. Ultimately, a relatively simple numerical model reflecting silica-based gel formation was developed and verified.

## Figures and Tables

**Table 1:** Designated model parameters: secondary particle concentration (*c*), probability of an effective collision (*P*) and time step (*dt*) for actual experimental syntheses A–F.

**A (1-2-0.9-0.9)**		**B (1-2-1-1)**		**C (1-2-1.1-1.1)**	
* 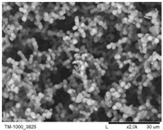 *	c=6.7% $P=1.4\times 10^{-4}$ $dt=1.34\times 10^{-1}$	* 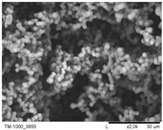 *	c=6.4% $P=1.8\times 10^{-4}$ $dt=1.31\times 10^{-1}$	* 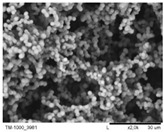 *	c=6.2% $P=1.9\times 10^{-4}$ $dt=1.14\times 10^{-1}$
**D (1-2-1.2-1.2)**		**E (1-2-1.3-1.3)**		**F (1-2-1.4-1.4)**	
* 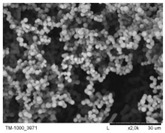 *	c=6% $P=2.8\times 10^{-4}$ $dt=1.08\times 10^{-1}$	* 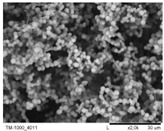 *	c=6.1% $P=3.5\times 10^{-4}$ $dt=1.12\times 10^{-1}$	* 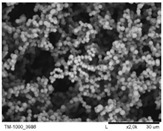 *	c=5.9% $P=3.1\times 10^{-4}$ $dt=1.02\times 10^{-1}$

**Table 2:** Condensation kinetics phases and mechanisms (experimental vs. numerical designation).

Condensation Kinetics Phases	Prevailing Mechanism of Structure Growth	
	Experimental	Numerical
1 nucleation	mechanism A	- (not considered in the model)
2 intensive mass growth (microscopic phase separation) phase	mechanism B	
3 cluster–cluster gelation (plateau)	mechanism C	

**Table 3:** Dependences of ${\left(\frac{dm}{dt}\right)}_{max}/t_{2}$ ratio on probability of an effective collision for (a) experimental, (b) numerical approach.

Approach	Established Dependence	Coefficient of Determination
(a) Experimental	$\frac{{\left(\frac{dm}{dt}\right)}_{max}}{t_{2}}=5.69\times 10^{-2}\;P-6.14\times 10^{-7}$	$R^{2}=0.87$
(b) Numerical	$\frac{{\left(\frac{dm}{dt}\right)}_{max}}{t_{2}}=4.37\times 10^{-2}\;P+7.07\times 10^{-7}$	$R^{2}=1$

**Table 4:** Comparison of our results of activation energy and the literature data.

Source	Precursor	$E\left[\frac{\mathrm{kJ}}{\mathrm{mol}}\right]\;$	Reference
This paper	methyltrimethoxysilane (MTMS)	2.6–37.6	-
A. Ponton et al. (2002)	tetramethoxysilane (TMOS)	37.1–38.2	[56]
C.J. Brinker and G.W. Scherer (1990)	general data for silica precursors	42–84	[13]

**Table 5:** Similarities and discrepancies between tendencies observed for experimental and numerical systems.

Parameter	Experimental	Numerical
	RLCA, $P=\langle 1.4\times 10^{-4},\;3.1\times 10^{-4}\rangle$ Narrow Range	RLCA–DLCA, $P=\langle 1\times 10^{-4},\;1\rangle$ Very Wide Range
$t_{1}$	Decreases (linearly) with an increase in the $n_{b}/n_{p}$ molar ratio Decreases with an increase in the probability (estimated based on Arrhenius eq.)	No data
$t_{2}$	Decreases (most likely in an exponential manner; power and linear functions are also a good approximation) with an increase in both the $n_{b}/n_{p}$ molar ratio and P.	Decrease in power function manner with the increase in probability.
$t_{g}$	Decreases with an increase in both $n_{b}/n_{p}$ and P. Exponential and power trendlines are a good fit. However, we use an exponential approximation for the designation of E, A	Decrease in power function of P.
$\frac{{\left(\frac{dm}{dt}\right)}_{max}}{t_{2}}$	Increases (but not constantly) with $n_{b}/n_{p}$ or P.	Seems to increase with concentration and P but in an unclear or constant manner.

**Table 6:** Chemical composition of the studied MTMS-based alcogel samples.

Synthesis Denotation	MTMS/MeOH Vol. Ratio	Acid/MTMS Vol. Ratio	Base/MTMS Vol. Ratio	Moles				
				MTMS	MeOH	Water	Acid	Base
A	1:2	0.9	0.9	0.0069	0.049	0.050	1.2 × 10^−9^	5.9 × 10^−8^
B		1.0	1.0			0.055	1.2 × 10^−9^	6.3 × 10^−8^
C		1.1	1.1			0.061	1.3 × 10^−9^	6.7 × 10^−8^
D		1.2	1.2			0.066	1.4 × 10^−9^	7.0 × 10^−8^
E		1.3	1.3			0.072	1.4 × 10^−9^	7.4 × 10^−8^
F		1.4	1.4			0.077	1.5 × 10^−9^	7.7 × 10^−8^

**Figure A1:**
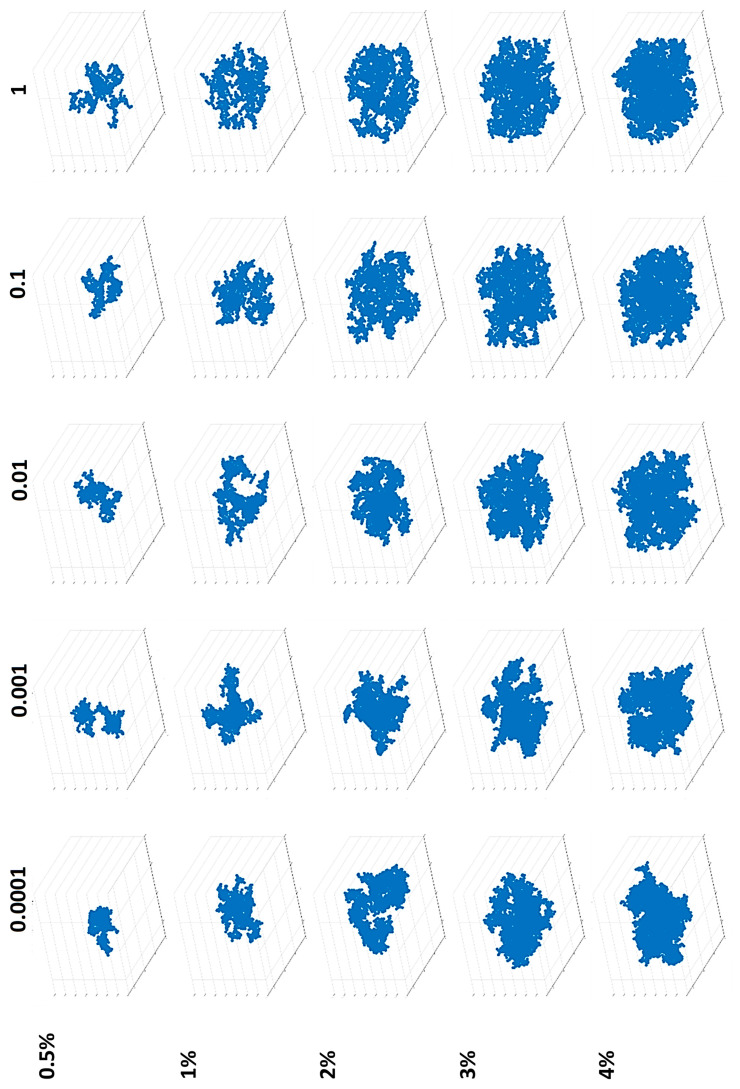
The numerically obtained structures for various secondary particle concentration (horizontal axis) and probability of an effective collision (vertical axis)—all 25 cases.

**Figure A2:**
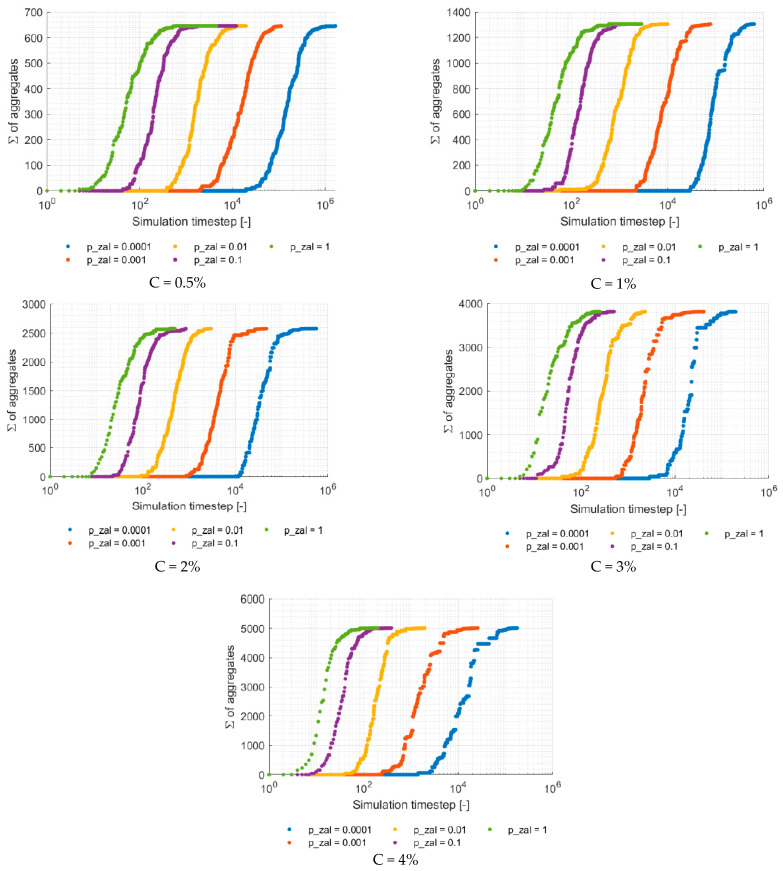
Kinetics curves obtained numerically for the all 25 cases.

## Data Availability

Not applicable.

## References

[1] Yamahara K., Okazaki K. (1998). Molecular Dynamics Simulation of the Structural Development in Sol-Gel Process for Silica Systems. Fluid Phase Equilib..

[2] Issa A.A., Luyt A.S. (2019). Kinetics of Alkoxysilanes and Organoalkoxysilanes Polymerization: A Review. Polymers.

[3] Innocenzi P. (2016). The Sol-to-Gel Transition.

[4] Dong H., Brook M.A., Brennan J.D. (2005). A New Route to Monolithic Methylsilsesquioxanes: Gelation Behavior of Methyltrimethoxysilane and Morphology of Resulting Methylsilsesquioxanes under One-Step and Two-Step Processing. Chem. Mater..

[5] Hüsing N., Schubert U. (1998). Aerogels—Airy Materials: Chemistry, Structure, and Properties. Angew. Chem. Int. Ed..

[6] Roy R. (1987). Ceramics by the Solution-Sol-Gel Route. Science.

[7] Deshmukh K., Kovářík T., Křenek T., Docheva D., Stich T., Pola J. (2020). Recent Advances and Future Perspectives of Sol-Gel Derived Porous Bioactive Glasses: A Review. RSC Adv..

[8] Brinker C.J., Hurd A.J., Schunk P.R., Frye G.C., Ashley C.S. (1992). Review of Sol-Gel Thin Film Formation. J. Non-Cryst. Solids.

[9] Minami T. (2013). Advanced Sol-Gel Coatings for Practical Applications. J. Sol-Gel Sci. Technol..

[10] Kloskowski A., Pilarczyk M., Chrzanowski W., Namieśnik J. (2010). Sol-Gel Technique—A Versatile Tool for Adsorbent Preparation. Crit. Rev. Anal. Chem..

[11] Jelle B.P., Baetens R., Gustavsen A. (2015). Aerogel Insulation for Building Applications. The Sol-Gel Handbook.

[12] Aegerter M.A., Leventis N., Koebel M.M. (2011). Aerogels Handbook.

[13] Brinker C.J., Scherer G.W. (1990). Sol-Gel Science The physics and Chemistry of Sol-Gel Processing.

[14] Nakanishi K., Kanamori K. (2005). Organic-Inorganic Hybrid Poly(Silsesquioxane) Monoliths with Controlled Macro- and Mesopores. J. Mater. Chem..

[15] Itagaki A., Nakanishi K., Hirao K. (2003). Phase Separation in Sol-Gel System Containing Mixture of 3- and 4-Functional Alkoxysilanes. J. Sol-Gel Sci. Technol..

[16] Kaji H., Nakanishi K., Soga N. (1993). Polymerization-Induced Phase Separation in Silica Sol-Gel Systems Containing Formamide. J. Sol-Gel Sci. Technol..

[17] Chateau D., Liotta A., Lundén H., Lerouge F., Chaput F., Krein D., Cooper T., Lopes C., El-Amay A.A.G., Lindgren M. (2016). Long Distance Enhancement of Nonlinear Optical Properties Using Low Concentration of Plasmonic Nanostructures in Dye Doped Monolithic Sol–Gel Materials. Adv. Funct. Mater..

[18] Pawlik N., Szpikowska-Sroka B., Goryczka T., Pisarski W.A. (2021). Studies of Sol-Gel Evolution and Distribution of Eu^3+^ Ions in Glass–Ceramics Containing Laf_3_ Nanocrystals Depending on Initial Sols Composition. Int. J. Mol. Sci..

[19] Barrulas R.V., López-Iglesias C., Zanatta M., Casimiro T., Mármol G., Carrott M.R., García-González C.A., Corvo M.C. (2022). The AEROPILs Generation: Novel Poly(Ionic Liquid)-based Aerogels for CO_2_ Capture. Int. J. Mol. Sci..

[20] Kubicka M., Bakierska M., Chudzik K., Świętosławski M., Molenda M. (2021). Nitrogen-Doped Carbon Aerogels Derived from Starch Biomass with Improved Electrochemical Properties for Li-Ion Batteries. Int. J. Mol. Sci..

[21] Babiarczuk B., Lewandowski D., Szczurek A., Kierzek K., Meffert M., Gerthsen D., Kaleta J., Krzak J. (2020). Novel Approach of Silica-PVA Hybrid Aerogel Synthesis by Simultaneous Sol-Gel Process and Phase Separation. J. Supercrit. Fluids.

[22] Pruna A., Cárcel A., Benedito A., Giménez E. (2021). Article Hydrothermal-Freeze-Casting of Poly(Amidoamine)-Modified Graphene Aerogels towards CO_2_ Adsorption. Int. J. Mol. Sci..

[23] Narayan R., Laberty-Robert C., Pelta J., Tarascon J.M., Dominko R. (2022). Self-Healing: An Emerging Technology for Next-Generation Smart Batteries. Adv. Energy Mater..

[24] Mo F., Li H., Pei Z., Liang G., Ma L., Yang Q., Wang D., Huang Y., Zhi C. (2018). A Smart Safe Rechargeable Zinc Ion Battery Based on Sol-Gel Transition Electrolytes. Sci. Bull..

[25] Fu L.J., Liu H., Li C., Wu Y.P., Rahm E., Holze R., Wu H.Q. (2005). Electrode Materials for Lithium Secondary Batteries Prepared by Sol-Gel Methods. Prog. Mater. Sci..

[26] Lim J., Malati P., Bonet F., Dunn B. (2007). Nanostructured Sol-Gel Electrodes for Biofuel Cells. J. Electrochem. Soc..

[27] Zhang S., Chen X., Du S., Wang J., Dong J., Wu D. (2021). Facile Synthesis of Highly Active Ti/Sb-SnO_2_ Electrode by Sol-Gel Spinning Technique for Landfill Leachate Treatment. Water Sci. Technol..

[28] Kong X., Li D., Fedorovskaya E.O., Kallio T., Ren X. (2021). New Insights in Al-Doping Effects on the LiNiO_2_ Positive Electrode Material by a Sol-Gel Method. Int. J. Energy Res..

[29] Reddy R.N., Reddy R.G. (2003). Sol-Gel MnO_2_ as an Electrode Material for Electrochemical Capacitors. J. Power Sources.

[30] Carvalho R.N.L., Cordas C.M., da Fonseca L.J.P. (2022). Electrode Kinetics of Ion Jelly and Ion Sol-Gel Redox Materials on Screen-Printed Electrodes. Appl. Sci..

[31] Ratke L., Hajduk A. (2015). On the Size Effect of Gelation Kinetics in Rf Aerogels. Gels.

[32] Ziff R.M., Ernst M.H., Hendriks E.M. (1983). Kinetics of Gelation and Universality. J. Phys. A Gen. Phys..

[33] Jiang Y., Gang H., Benkun M. (1990). Critical Property and Universality in the Generalised Smoluchovski Coagulation Equation. Phys. Rev. B.

[34] Mellema M., van Opheusden J.H.J., van Vliet T. (1999). Relating Colloidal Particle Interactions to Gel Structure Using Brownian Dynamics Simulations and the Fuchs Stability Ratio. J. Chem. Phys..

[35] Whittle M., Dickinson E. (1997). Brownian Dynamics Simulation of Gelation in Soft Sphere Systems with Irreversible Bond Formation. Mol. Phys..

[36] Gelb L.D. (2007). Simulating Silica Aerogels with a Coarse-Grained Flexible Model and Langevin Dynamics. J. Phys. Chem. C.

[37] Ferreiro-Rangel C.A., Gelb L.D. (2013). Investigation of the Bulk Modulus of Silica Aerogel Using Molecular Dynamics Simulations of a Coarse-Grained Model. J. Phys. Chem. B.

[38] Depta P.N., Gurikov P., Schroeter B., Forgács A., Kalmár J., Paul G., Marchese L., Heinrich S., Dosta M. (2022). DEM-Based Approach for the Modeling of Gelation and Its Application to Alginate. J. Chem. Inf. Model..

[39] Martin J.E., Wilcoxon J., Adolf D. (1987). Critical Exponents for the Sol-Gel Transition. Phys. Rev. A.

[40] Shibayama M. (2002). Gel Formation Analyses by Dynamic Light Scattering. Bull. Chem. Soc. Jpn..

[41] Hsieh K., Lallet F., Olivi-Tran N. (2008). DLCA and Langevin Dynamics Approaches of Sol Gel Transition: A Comparison via the Fractal Dimension during Aggregation. Fractals.

[42] Abdusalamabov R., Itskov M., Milow B., Reichenauer G., Rege A. Investigation of the Fractal Properties of Aerogels by Di Usion-Limited Aggregation Models. Proceedings of the 8th GACM Colloquium on Computational Mechanics: For Young Scientists from Academia and Industry.

[43] Weitz D.A., Huang J.S., Lin M.Y., Sung J. (1984). Dynamics of Diffusion-Limited Kinetic Aggregation. Phys. Rev. Lett..

[44] Borzęcka N.H., Kozłowska I., Gac J.M., Bojarska M. (2019). Anti-Fouling Properties of Poly(Acrylic Acid) Grafted Ultrafiltration Membranes—Experimental and Theoretical Study. Appl. Surf. Sci..

[45] Borzęcka N.H., Nowak B., Pakuła R., Przewodzki R., Gac J.M. (2021). Cellular Automata Modeling of Silica Aerogel Condensation Kinetics. Gels.

[46] Morales R.V., da Cunha C.R., Rambo C.R. (2014). A Complex Network Approach for the Growth of Aerogels. Phys. A Stat. Mech. Its Appl..

[47] Garofalini S.H., Martin G. (1994). Molecular Simulations of the Polymerization of Silicic Acid Molecules and Network Formation. J. Phys. Chem..

[48] Pereira J.C.G., Catlow C.R.A., Price G.D. (2002). Molecular Dynamics Simulation of Methanolic and Ethanolic Silica-Based Sol-Gel Solutions at Ambient Temperature and Pressure. J. Phys. Chem. A.

[49] Elanany M., Selvam P., Yokosuka T., Takami S., Kubo M., Imamura A., Miyamoto A. (2003). A Quantum Molecular Dynamics Simulation Study of the Initial Hydrolysis Step in Sol-Gel Process. J. Phys. Chem. B.

[50] Bałdyga J., Tyl G., Bouaifi M. (2019). Aggregation Efficiency of Amorphous Silica Nanoparticles. Chem. Eng. Technol..

[51] Aniszewska D., Rybaczuk M. (2021). Mechanical Properties of Silica Aerogels Modelled by Movable Cellular Automata Simulations. Mater. Today Commun..

[52] Smolin A.Y., Shilko E.V., Astafurov S.V., Konovalenko I.S., Buyakova S.P., Psakhie S.G. (2015). Modeling Mechanical Behaviors of Composites with Various Ratios of Matrix–Inclusion Properties Using Movable Cellular Automaton Method. Def. Technol..

[53] Scalise D., Schulman R. (2016). Emulating Cellular Automata in Chemical Reaction–Diffusion Networks. Nat. Comput..

[54] Davies C.H.J. (1997). Growth of Nuclei in a Cellular Automaton Simulation of Recrystallisation. Scr. Mater..

[55] Wessling B. (1996). Cellular Automata Simulation of Dissipative Structure Formation in Heterogeneous Polymer Systems, Formation of Networks of a Dispersed Phase by Flocculation. J. Phys. II.

[56] Ponton A., Warlus S., Griesmar P. (2002). Rheological Study of the Sol-Gel Transition in Silica Alkoxides. J. Colloid Interface Sci..

[57] Rao A.V., Bhagat S.D., Hirashima H., Pajonk G. (2006). Synthesis of flexible silica aerogels using methyltrimethoxysilane (MTMS) precursor. J. Colloid Interface Sci..

[58] Boonstra A.H., Bernards T.N.M. (1988). The Dependence of the Gelation Time on the Hydrolysis Time in a Two-Step SiO_2_ Sol-Gel Process. J. Non-Cryst. Solids.

[59] Taylor S.J., Haw M.D., Sefcik J., Fletcher A.J. (2014). Monitoring the Gelation Mechanism of Resorcinol-Formaldehyde Gels by Dynamic Light Scattering. Langmuir.

[60] Gaca K.Z., Sefcik J. (2013). Mechanism and Kinetics of Nanostructure Evolution during Early Stages of Resorcinol-Formaldehyde Polymerisation. J. Colloid Interface Sci..

[61] Borzęcka N.H., Nowak B., Gac J.M., Głaz T., Bojarska M. (2020). Kinetics of MTMS-Based Aerogel Formation by the Sol-Gel Method—Experimental Results and Theoretical Description. J. Non-Cryst. Solids.

[62] Maleki H. (2016). Recent Advances in Aerogels for Environmental Remediation Applications. Chem. Eng. J..

[63] Kier L.B., Seybold P.G., Cheng C.-K. (2005). Cellular Automata Modeling of Chemical Systems a Textbook and Laboratory Manual.

